# 4-[3-(4-Fluoro­phen­yl)quinoxalin-2-yl]-*N*-isopropyl­pyridin-2-amine

**DOI:** 10.1107/S1600536809018285

**Published:** 2009-05-20

**Authors:** Pierre Koch, Dieter Schollmeyer, Stefan Laufer

**Affiliations:** a Institute of Pharmacy, Department of Pharmaceutical and Medicinal Chemistry, Eberhard-Karls-University Tübingen, Auf der Morgenstelle 8, 72076 Tübingen, Germany; b Department of Organic Chemistry, Johannes Gutenberg-University Mainz, Duesbergweg 10-14, D-55099 Mainz, Germany

## Abstract

In the crystal structure of the title compound, C_22_H_19_FN_4_, the quinoxaline system makes dihedral angles of 32.07 (13) and 69.64 (13)° with the 4-fluoro­phenyl and pyridine rings, respectively. The 4-fluoro­phenyl ring makes a dihedral angle of 71.77 (16)° with the pyridine ring. The crystal structure is stabilized by inter­molecular N—H⋯N hydrogen bonding.

## Related literature

For chinoxaline derivatives and their biological activity, see: He *et al.* (2003 ▶); Kim *et al.* (2004 ▶).
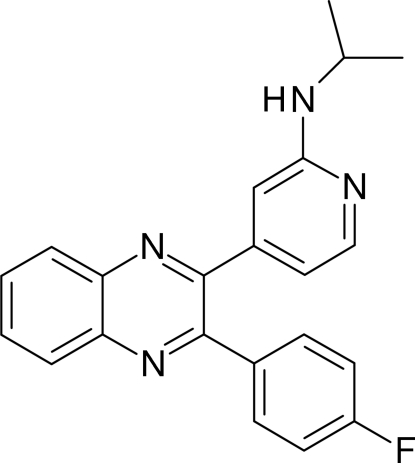


## Experimental

### 

#### Crystal data

C_22_H_19_FN_4_
*M* *_r_* = 358.41
Monoclinic, 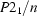

*a* = 17.230 (9) Å
*b* = 5.386 (3) Å
*c* = 19.123 (10) Å
β = 96.114 (13)°
*V* = 1764.4 (16) Å^3^
*Z* = 4
Mo *K*α radiation
μ = 0.09 mm^−1^
*T* = 173 K
0.4 × 0.06 × 0.03 mm

#### Data collection

Bruker SMART CCD diffractometer
Absorption correction: none
20392 measured reflections
4217 independent reflections
1201 reflections with *I* > 2σ(*I*)
*R*_int_ = 0.236

#### Refinement

*R*[*F* ^2^ > 2σ(*F* ^2^)] = 0.049
*wR*(*F* ^2^) = 0.123
*S* = 0.74
4217 reflections
247 parameters
H-atom parameters constrained
Δρ_max_ = 0.23 e Å^−3^
Δρ_min_ = −0.22 e Å^−3^

### 

Data collection: *APEX2* (Bruker, 2006 ▶); cell refinement: *SAINT* (Bruker, 2006 ▶); data reduction: *SAINT*; program(s) used to solve structure: *SIR97* (Altomare *et al.*, 1999 ▶); program(s) used to refine structure: *SHELXL97* (Sheldrick, 2008 ▶); molecular graphics: *PLATON* (Spek, 2009 ▶); software used to prepare material for publication: *PLATON*.

## Supplementary Material

Crystal structure: contains datablocks I, global. DOI: 10.1107/S1600536809018285/bt2957sup1.cif

Structure factors: contains datablocks I. DOI: 10.1107/S1600536809018285/bt2957Isup2.hkl

Additional supplementary materials:  crystallographic information; 3D view; checkCIF report

## Figures and Tables

**Table 1:** Hydrogen-bond geometry (Å, °)

*D*—H⋯*A*	*D*—H	H⋯*A*	*D*⋯*A*	*D*—H⋯*A*
N17—H17⋯N14^i^	1.01	2.16	3.137 (4)	162

Symmetry code: (i) 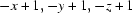.

**Fig. 1.**
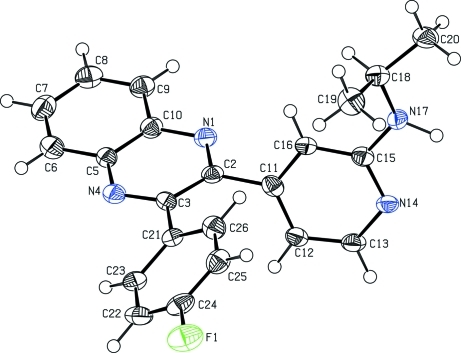
View of compound I. Displacement ellipsoids are drawn at the 50% probability level. H atoms are depicted as circles of arbitrary size.

**Fig. 2.**
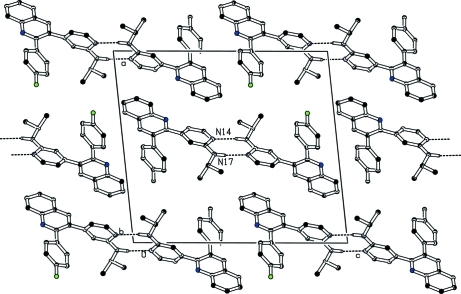
Part of the crystal packing of compound I. The hydrogen bond is shown with dashed lines. View along b axis.

## supplementary crystallographic information

### Comment

Functionalized quinoxaline derivatives are well known in pharmaceutical industry. They have been shown to possess antibacterial activity (Kim *et al*. 2004) and as PDGF-*R* tyrosine kinase inhibitor (He *et al.* 2003).

The title compound, 4-(3-(4-fluorophenyl)quinoxalin-2-yl)-*N*- isopropylpyridin-2-amine (I), was prepared in the course of our studies on 2-(2-alkylaminopyridin-4-yl)-3-(4-fluorophenyl)quinoxalines as potent p38 mitogen-activated protein (MAP) kinase inhibitors.

The analysis of the crystal structure of compound I is shown in Figure 1. As might be expected the 4-fluorophenyl, the pyridine ring as well as the quinoxaline ring are planar. The quinoxaline ring makes dihedral angles of 32.07 (13)° and 69.64 (13)° to the 4-fluorophenyl ring and the pyridine ring, respectively. The 4-fluorophenyl ring makes dihedral angles of 71.77 (16)° to the pyridine ring.

The crystal packing (Figure 2) shows that N17—H17 of the imidazole ring forms an intermolecular N–H···N hydrogen bond to pyridine (N14) resulting in a dimer. The length of the hydrogen bond is 2.16Å (Table 1).

### Experimental

*tert*-Butyl 4-(3-(4-fluorophenyl)quinoxalin-2-yl)pyridin-2-yl(isopropyl)carbamate (120 mg, 0.26 mmol) was dissolved in DCM (2 ml), treated with trifluoroacetic acid (2 ml) and stirred for 16 h at 298 K. The reaction mixture was cooled to 273 K and neutralized with 1 N aqueous NaOH-solution to pH 12. Ethyl acetate was added and the organic layer was washed with water, dried over sodium sulfate and the solvent was removed under reduced pressure. The crude product was purified by flash-chromatography (silica gel, petroleum ether - ethyl acetate 4–1 to 2–1) to yield the title compound I (78 mg, 84%) as a colourless solid. The compound was recrystalized from dimethylsulfoxide.

### Refinement

Hydrogen atoms attached to carbons were placed at calculated positions with C—H = 0.95 Å (aromatic) or 0.98–0.99 Å (*sp*^3^ C-atom). They were refined in the riding-model approximation with isotropic displacement parameters (set at 1.2–1.5 times of the *U*_eq_ of the parent atom). The hydrogen atom attached to N17 was located in difference Fourier maps and freely refined.

### Crystal data

**Table:**

C_22_H_19_FN_4_	*F*(000) = 752
*M**_r_* = 358.41	*D*_x_ = 1.349 Mg m^−^^3^
Monoclinic, *P*2_1_/*n*	Mo *K*α radiation, λ = 0.71073 Å
Hall symbol: -P 2yn	Cell parameters from 934 reflections
*a* = 17.230 (9) Å	θ = 2.2–19.5°
*b* = 5.386 (3) Å	µ = 0.09 mm^−^^1^
*c* = 19.123 (10) Å	*T* = 173 K
β = 96.114 (13)°	Needle, colourless
*V* = 1764.4 (16) Å^3^	0.4 × 0.06 × 0.03 mm
*Z* = 4	

### Data collection

**Table:**

Bruker SMART CCD diffractometer	1201 reflections with *I* > 2σ(*I*)
Radiation source: sealed Tube	*R*_int_ = 0.236
graphite	θ_max_ = 28.0°, θ_min_ = 1.5°
CCD scans	*h* = −22→21
20392 measured reflections	*k* = −7→6
4217 independent reflections	*l* = −25→25

### Refinement

**Table:**

Refinement on *F*^2^	Secondary atom site location: difference Fourier map
Least-squares matrix: full	Hydrogen site location: inferred from neighbouring sites
*R*[*F*^2^ > 2σ(*F*^2^)] = 0.049	H-atom parameters constrained
*wR*(*F*^2^) = 0.123	*w* = 1/[σ^2^(*F*_o_^2^) + (0.03*P*)^2^] where *P* = (*F*_o_^2^ + 2*F*_c_^2^)/3
*S* = 0.74	(Δ/σ)_max_ < 0.001
4217 reflections	Δρ_max_ = 0.23 e Å^−^^3^
247 parameters	Δρ_min_ = −0.22 e Å^−^^3^
0 restraints	Extinction correction: *SHELXL97* (Sheldrick, 2008), Fc^*^=kFc[1+0.001xFc^2^λ^3^/sin(2θ)]^-1/4^
Primary atom site location: structure-invariant direct methods	Extinction coefficient: 0.0111 (10)

### Special details

**Table:**

Geometry. All e.s.d.'s (except the e.s.d. in the dihedral angle between two l.s. planes) are estimated using the full covariance matrix. The cell e.s.d.'s are taken into account individually in the estimation of e.s.d.'s in distances, angles and torsion angles; correlations between e.s.d.'s in cell parameters are only used when they are defined by crystal symmetry. An approximate (isotropic) treatment of cell e.s.d.'s is used for estimating e.s.d.'s involving l.s. planes.
Refinement. Refinement of *F*^2^ against ALL reflections. The weighted *R*-factor *wR* and goodness of fit *S* are based on *F*^2^, conventional *R*-factors *R* are based on *F*, with *F* set to zero for negative *F*^2^. The threshold expression of *F*^2^ > σ(*F*^2^) is used only for calculating *R*-factors(gt) *etc*. and is not relevant to the choice of reflections for refinement. *R*-factors based on *F*^2^ are statistically about twice as large as those based on *F*, and *R*- factors based on ALL data will be even larger.

### Fractional atomic coordinates and isotropic or equivalent isotropic displacement parameters (Å^2^)

**Table:**

	*x*	*y*	*z*	*U*_iso_*/*U*_eq_	
F1	0.32320 (13)	−0.2743 (4)	0.12436 (11)	0.0534 (7)	
N1	0.64239 (16)	0.7424 (5)	0.21100 (13)	0.0280 (7)	
C2	0.5913 (2)	0.5582 (6)	0.20994 (17)	0.0247 (9)	
C3	0.5637 (2)	0.4311 (6)	0.14642 (17)	0.0247 (9)	
N4	0.59317 (17)	0.4791 (5)	0.08672 (14)	0.0286 (8)	
C5	0.6482 (2)	0.6633 (6)	0.08760 (18)	0.0261 (9)	
C6	0.6806 (2)	0.7247 (7)	0.02532 (18)	0.0354 (9)	
H6	0.6676	0.6298	−0.0162	0.043*	
C7	0.7308 (2)	0.9216 (7)	0.02466 (19)	0.0391 (10)	
H7	0.7523	0.9632	−0.0176	0.047*	
C8	0.7511 (2)	1.0634 (7)	0.08587 (19)	0.0360 (10)	
H8	0.7853	1.2010	0.0843	0.043*	
C9	0.7220 (2)	1.0048 (6)	0.14717 (18)	0.0320 (10)	
H9	0.7362	1.0994	0.1885	0.038*	
C10	0.6703 (2)	0.8013 (6)	0.14864 (18)	0.0288 (9)	
C11	0.5688 (2)	0.4924 (6)	0.28109 (17)	0.0266 (9)	
C12	0.5957 (2)	0.2739 (6)	0.31406 (17)	0.0283 (9)	
H12	0.6229	0.1531	0.2899	0.034*	
C13	0.5814 (2)	0.2386 (6)	0.38249 (17)	0.0299 (9)	
H13	0.6023	0.0934	0.4056	0.036*	
N14	0.54053 (16)	0.3921 (5)	0.41920 (13)	0.0260 (7)	
C15	0.50848 (19)	0.5941 (6)	0.38430 (17)	0.0259 (9)	
C16	0.5247 (2)	0.6521 (6)	0.31639 (17)	0.0271 (9)	
H16	0.5054	0.8016	0.2946	0.033*	
N17	0.46189 (16)	0.7389 (5)	0.42120 (13)	0.0289 (7)	
H17	0.4566	0.6623	0.4685	0.035*	
C18	0.4038 (2)	0.9131 (6)	0.38765 (17)	0.0309 (9)	
H18	0.4304	1.0266	0.3564	0.037*	
C19	0.3386 (2)	0.7750 (7)	0.34280 (18)	0.0419 (10)	
H19A	0.3128	0.6598	0.3726	0.063*	
H19B	0.3609	0.6818	0.3057	0.063*	
H19C	0.3004	0.8949	0.3214	0.063*	
C20	0.3708 (2)	1.0676 (6)	0.44350 (17)	0.0374 (10)	
H20A	0.3427	0.9596	0.4735	0.056*	
H20B	0.3347	1.1917	0.4210	0.056*	
H20C	0.4134	1.1516	0.4722	0.056*	
C21	0.4997 (2)	0.2437 (6)	0.14209 (16)	0.0255 (8)	
C22	0.4995 (2)	0.0513 (6)	0.09329 (17)	0.0301 (9)	
H22	0.5402	0.0418	0.0636	0.036*	
C23	0.4409 (2)	−0.1263 (6)	0.08723 (18)	0.0356 (10)	
H23	0.4414	−0.2598	0.0548	0.043*	
C24	0.3825 (2)	−0.1020 (7)	0.1296 (2)	0.0360 (10)	
C25	0.3785 (2)	0.0856 (7)	0.17772 (18)	0.0347 (10)	
H25	0.3365	0.0952	0.2060	0.042*	
C26	0.4380 (2)	0.2604 (7)	0.18368 (17)	0.0321 (9)	
H26	0.4367	0.3928	0.2164	0.039*	

### Atomic displacement parameters (Å^2^)

**Table:**

	*U*^11^	*U*^22^	*U*^33^	*U*^12^	*U*^13^	*U*^23^
F1	0.0456 (16)	0.0392 (14)	0.0736 (16)	−0.0154 (12)	−0.0022 (12)	0.0043 (13)
N1	0.0310 (19)	0.0222 (16)	0.0312 (17)	0.0018 (16)	0.0047 (14)	0.0014 (15)
C2	0.025 (2)	0.020 (2)	0.030 (2)	0.0058 (17)	0.0039 (18)	0.0025 (18)
C3	0.027 (2)	0.022 (2)	0.026 (2)	0.0052 (17)	0.0060 (18)	−0.0013 (18)
N4	0.0292 (19)	0.0244 (18)	0.0325 (18)	0.0068 (15)	0.0053 (15)	0.0005 (14)
C5	0.028 (2)	0.024 (2)	0.027 (2)	0.0018 (17)	0.0059 (18)	0.0003 (17)
C6	0.037 (3)	0.033 (2)	0.037 (2)	0.005 (2)	0.0078 (19)	−0.002 (2)
C7	0.038 (3)	0.040 (3)	0.040 (3)	0.007 (2)	0.012 (2)	0.011 (2)
C8	0.031 (3)	0.030 (2)	0.047 (2)	0.0018 (18)	0.005 (2)	0.004 (2)
C9	0.029 (2)	0.029 (2)	0.038 (2)	0.0020 (18)	0.0052 (19)	0.0032 (18)
C10	0.028 (2)	0.027 (2)	0.032 (2)	0.0022 (18)	0.0067 (18)	0.0028 (18)
C11	0.026 (2)	0.025 (2)	0.029 (2)	−0.0075 (17)	0.0027 (18)	−0.0067 (18)
C12	0.033 (2)	0.021 (2)	0.030 (2)	0.0001 (18)	0.0025 (18)	−0.0038 (18)
C13	0.032 (2)	0.020 (2)	0.039 (2)	0.0047 (19)	0.0096 (19)	0.0011 (19)
N14	0.0308 (19)	0.0169 (16)	0.0307 (17)	−0.0021 (15)	0.0051 (15)	−0.0004 (14)
C15	0.026 (2)	0.022 (2)	0.030 (2)	−0.0021 (18)	0.0031 (18)	−0.0024 (18)
C16	0.035 (2)	0.018 (2)	0.028 (2)	−0.0004 (16)	0.0027 (19)	−0.0013 (16)
N17	0.0362 (19)	0.0258 (17)	0.0254 (16)	0.0099 (15)	0.0060 (14)	0.0040 (15)
C18	0.032 (2)	0.025 (2)	0.035 (2)	−0.0007 (19)	0.0040 (19)	−0.0001 (18)
C19	0.037 (3)	0.046 (3)	0.042 (2)	0.001 (2)	0.001 (2)	−0.002 (2)
C20	0.041 (3)	0.029 (2)	0.044 (2)	0.0097 (19)	0.014 (2)	0.0001 (19)
C21	0.029 (2)	0.0200 (19)	0.0268 (19)	0.0030 (19)	0.0021 (17)	0.0039 (18)
C22	0.033 (2)	0.024 (2)	0.033 (2)	0.0069 (18)	0.0013 (19)	0.0042 (19)
C23	0.041 (3)	0.021 (2)	0.043 (2)	0.0047 (19)	−0.006 (2)	−0.0029 (18)
C24	0.035 (3)	0.026 (2)	0.045 (2)	−0.011 (2)	−0.003 (2)	0.009 (2)
C25	0.031 (3)	0.037 (2)	0.035 (2)	0.003 (2)	0.0031 (19)	0.006 (2)
C26	0.031 (2)	0.033 (2)	0.032 (2)	0.001 (2)	0.0004 (18)	−0.0017 (19)

### Geometric parameters (Å, °)

**Table:**

F1—C24	1.376 (4)	C15—N17	1.368 (4)
N1—C2	1.325 (4)	C15—C16	1.393 (4)
N1—C10	1.370 (4)	C16—H16	0.9500
C2—C3	1.431 (4)	N17—C18	1.469 (4)
C2—C11	1.497 (4)	N17—H17	1.0071
C3—N4	1.324 (4)	C18—C20	1.512 (4)
C3—C21	1.491 (4)	C18—C19	1.532 (4)
N4—C5	1.371 (4)	C18—H18	1.0000
C5—C10	1.402 (4)	C19—H19A	0.9800
C5—C6	1.407 (4)	C19—H19B	0.9800
C6—C7	1.370 (5)	C19—H19C	0.9800
C6—H6	0.9500	C20—H20A	0.9800
C7—C8	1.410 (4)	C20—H20B	0.9800
C7—H7	0.9500	C20—H20C	0.9800
C8—C9	1.360 (4)	C21—C22	1.394 (4)
C8—H8	0.9500	C21—C26	1.397 (4)
C9—C10	1.414 (4)	C22—C23	1.386 (5)
C9—H9	0.9500	C22—H22	0.9500
C11—C16	1.372 (4)	C23—C24	1.363 (4)
C11—C12	1.391 (4)	C23—H23	0.9500
C12—C13	1.370 (4)	C24—C25	1.373 (4)
C12—H12	0.9500	C25—C26	1.387 (4)
C13—N14	1.334 (4)	C25—H25	0.9500
C13—H13	0.9500	C26—H26	0.9500
N14—C15	1.362 (4)		
			
C2—N1—C10	117.0 (3)	C15—C16—H16	120.2
N1—C2—C3	122.1 (3)	C15—N17—C18	123.3 (3)
N1—C2—C11	113.5 (3)	C15—N17—H17	110.1
C3—C2—C11	124.3 (3)	C18—N17—H17	122.1
N4—C3—C2	121.0 (3)	N17—C18—C20	109.5 (3)
N4—C3—C21	115.7 (3)	N17—C18—C19	111.1 (3)
C2—C3—C21	123.2 (3)	C20—C18—C19	110.7 (3)
C3—N4—C5	117.3 (3)	N17—C18—H18	108.5
N4—C5—C10	121.4 (3)	C20—C18—H18	108.5
N4—C5—C6	119.7 (3)	C19—C18—H18	108.5
C10—C5—C6	118.8 (3)	C18—C19—H19A	109.5
C7—C6—C5	119.9 (3)	C18—C19—H19B	109.5
C7—C6—H6	120.1	H19A—C19—H19B	109.5
C5—C6—H6	120.1	C18—C19—H19C	109.5
C6—C7—C8	120.8 (3)	H19A—C19—H19C	109.5
C6—C7—H7	119.6	H19B—C19—H19C	109.5
C8—C7—H7	119.6	C18—C20—H20A	109.5
C9—C8—C7	120.6 (4)	C18—C20—H20B	109.5
C9—C8—H8	119.7	H20A—C20—H20B	109.5
C7—C8—H8	119.7	C18—C20—H20C	109.5
C8—C9—C10	119.2 (4)	H20A—C20—H20C	109.5
C8—C9—H9	120.4	H20B—C20—H20C	109.5
C10—C9—H9	120.4	C22—C21—C26	118.8 (3)
N1—C10—C5	120.8 (3)	C22—C21—C3	119.3 (3)
N1—C10—C9	118.5 (3)	C26—C21—C3	121.9 (3)
C5—C10—C9	120.7 (3)	C23—C22—C21	121.2 (3)
C16—C11—C12	118.8 (3)	C23—C22—H22	119.4
C16—C11—C2	120.6 (3)	C21—C22—H22	119.4
C12—C11—C2	120.4 (3)	C24—C23—C22	117.4 (3)
C13—C12—C11	117.7 (3)	C24—C23—H23	121.3
C13—C12—H12	121.1	C22—C23—H23	121.3
C11—C12—H12	121.1	C23—C24—C25	124.2 (4)
N14—C13—C12	125.1 (3)	C23—C24—F1	118.8 (4)
N14—C13—H13	117.4	C25—C24—F1	117.0 (4)
C12—C13—H13	117.4	C24—C25—C26	117.7 (4)
C13—N14—C15	116.5 (3)	C24—C25—H25	121.1
N14—C15—N17	115.6 (3)	C26—C25—H25	121.1
N14—C15—C16	121.6 (3)	C25—C26—C21	120.6 (4)
N17—C15—C16	122.7 (3)	C25—C26—H26	119.7
C11—C16—C15	119.7 (3)	C21—C26—H26	119.7
C11—C16—H16	120.2		
			
C10—N1—C2—C3	−2.4 (5)	C2—C11—C12—C13	171.9 (3)
C10—N1—C2—C11	175.3 (3)	C11—C12—C13—N14	3.4 (5)
N1—C2—C3—N4	5.8 (5)	C12—C13—N14—C15	2.5 (5)
C11—C2—C3—N4	−171.7 (3)	C13—N14—C15—N17	175.2 (3)
N1—C2—C3—C21	−172.7 (3)	C13—N14—C15—C16	−6.9 (5)
C11—C2—C3—C21	9.9 (5)	C12—C11—C16—C15	0.6 (5)
C2—C3—N4—C5	−3.1 (5)	C2—C11—C16—C15	−176.1 (3)
C21—C3—N4—C5	175.4 (3)	N14—C15—C16—C11	5.5 (5)
C3—N4—C5—C10	−2.2 (5)	N17—C15—C16—C11	−176.8 (3)
C3—N4—C5—C6	−179.0 (3)	N14—C15—N17—C18	−160.1 (3)
N4—C5—C6—C7	174.8 (3)	C16—C15—N17—C18	22.0 (5)
C10—C5—C6—C7	−2.1 (5)	C15—N17—C18—C20	−172.2 (3)
C5—C6—C7—C8	0.4 (5)	C15—N17—C18—C19	65.2 (4)
C6—C7—C8—C9	1.0 (6)	N4—C3—C21—C22	31.4 (5)
C7—C8—C9—C10	−0.7 (5)	C2—C3—C21—C22	−150.1 (3)
C2—N1—C10—C5	−2.9 (5)	N4—C3—C21—C26	−146.0 (3)
C2—N1—C10—C9	177.0 (3)	C2—C3—C21—C26	32.5 (5)
N4—C5—C10—N1	5.5 (5)	C26—C21—C22—C23	−2.2 (5)
C6—C5—C10—N1	−177.7 (3)	C3—C21—C22—C23	−179.7 (3)
N4—C5—C10—C9	−174.4 (3)	C21—C22—C23—C24	1.6 (5)
C6—C5—C10—C9	2.4 (5)	C22—C23—C24—C25	−0.3 (6)
C8—C9—C10—N1	179.1 (3)	C22—C23—C24—F1	179.5 (3)
C8—C9—C10—C5	−1.0 (5)	C23—C24—C25—C26	−0.3 (5)
N1—C2—C11—C16	69.2 (4)	F1—C24—C25—C26	179.9 (3)
C3—C2—C11—C16	−113.1 (4)	C24—C25—C26—C21	−0.3 (5)
N1—C2—C11—C12	−107.4 (4)	C22—C21—C26—C25	1.5 (5)
C3—C2—C11—C12	70.3 (5)	C3—C21—C26—C25	178.9 (3)
C16—C11—C12—C13	−4.8 (5)		

### Hydrogen-bond geometry (Å, °)

**Table:**

*D*—H···*A*	*D*—H	H···*A*	*D*···*A*	*D*—H···*A*
N17—H17···N14^i^	1.01	2.16	3.137 (4)	162

Symmetry codes: (i) −*x*+1, −*y*+1, −*z*+1.
